# PATIENT-CENTERED OUTCOME MEASUREMENT IN NURSING HOME-BASED PRAGMATIC TRIALS: CAPTURING WHAT COUNTS

**DOI:** 10.1093/geroni/igae098.1373

**Published:** 2024-12-31

**Authors:** Ellen McCreedy, Anthony Sisti, Roee Gutman, Rosa Baier, Ann Reddy, Vincent Mor

**Affiliations:** Veterans’ Administration, Providence, Rhode Island, United States; Brown University, Providence, Rhode Island, United States; Brown University, Providence, Rhode Island, United States; Brown University, Providence, Rhode Island, United States; Brown University School of Public Health, Providence, Rhode Island, United States; Brown University, Providence, Rhode Island, United States

## Abstract

Embedded pragmatic randomized controlled trials (ePCTs) rely on routinely collected data to evaluate study outcomes. Using data from Music & MEmory: A Pragmatic TRial for Nursing Home Residents With ALzheimer’s Disease (METRIcAL), we highlight the strengths and weaknesses of using routinely collected data to evaluate agitated behaviors in nursing home (NH) residents with dementia. We conducted two National Institute on Aging-funded ePCTs, one enrolled 976 residents (483 treatment, 493 control) from 54 NHs between June, 2019 and March, 2020, and the other enrolled 850 residents (431 Treatment, 419 control) from 54 NHs between May, 2021 and September, 2022. In both trials, there was no effect of the intervention on agitated behaviors, as measured by the Minimum Data Set (MDS), a routinely collected, pragmatic source of data in NHs. The intervention also did not affect behaviors as reported by staff using the Cohen-Mansfield Agitation Inventory (CMAI). However, we found a reduction in the frequency of nonaggressive agitated behaviors in response to the personalized music intervention, using the Agitation Behavior Mapping Instrument to conduct structured observations of residents, at standardized times of day, in treatment and control NHs. A personalized music intervention for NH residents with dementia reduced nonaggressive agitation, but these changes were not captured in the routinely collected administrative data (MDS) or by interviewing staff using the CMAI. Temporary reprieve from agitated behaviors may improve the quality of life for persons living with dementia, but these effects are not captured using pragmatic data or tools that rely on staff recall.

